# Tanshinone IIA Protects Ischemia/Reperfusion-Induced Cardiomyocyte Injury by Inhibiting the *HAS2*/*FGF9* Axis

**DOI:** 10.1155/2024/2581638

**Published:** 2024-11-13

**Authors:** Yanzhe Wang, Weixin Sun, Le Shen, Peng Yu, Qiusheng Shen, Yaozhong Zhou, Lu Yao, Xiaohu Chen

**Affiliations:** ^1^Department of Cardiology, Changshu Hospital Affiliated to Nanjing University of Chinese Medicine, Changshu, China; ^2^First Clinical Medical College, Nanjing University of Chinese Medicine, Nanjing, China; ^3^Yancheng TCM Hospital Affiliated to Nanjing University of Chinese Medicine, Yancheng TCM Hospital, Yancheng, China; ^4^Department of Cardiology, Jiangsu Province Hospital of Chinese Medicine, Affiliated Hospital of Nanjing University of Chinese Medicine, Nanjing, China

## Abstract

**Purpose:**

This study aimed to investigate the impacts of tanshinone IIA (Tan IIA) on ischemia/reperfusion (I/R)-induced cardiomyocyte injury in coronary heart disease (CHD), and to determine whether Tan IIA regulates myocardial cell injury induced by I/R through the Hyaluronan Synthase 2*/*fibroblast growth factor 9 *(HAS2/FGF9)* axis.

**Methods:**

Weighted gene co-expression network analysis (WGCNA) of the GSE23561 microarray dataset determined gene modules linked to CHD. The key genes were further explored through differential expression and protein-protein interaction (PPI) network analyses. Human AC16 cardiomyocytes were treated with Tan IIA, *HAS2* knockdown, and *FGF9* overexpression and they were exposed to normoxic, hypoxic, and I/R environments. Cell viability, apoptosis, gene/protein expression, and markers of oxidative stress were evaluated *in vitro*.

**Results:**

The turquoise module was significantly correlated with CHD and *HAS2* was identified as a hub gene. Under hypoxic conditions, Tan IIA exhibited a dose-dependent cardioprotective effect. Tan IIA ameliorated I/R-induced cellular injury, as evidenced by increased cell viability, decreased apoptosis, and regulation of key proteins (PCNA, Bax). After I/R conditions, knockdown of *HAS2* increased cell viability and reduced apoptosis, whereas overexpression of *FGF9* reversed these effects. Notably, *HAS2* knockdown also ameliorated I/R-induced increases in inflammatory cytokines and oxidative stress, and synergistic protection was provided by combined treatment with *FGF9* and Tan IIA.

**Conclusion:**

Taken together, our findings confirm that Tan IIA protects cardiomyocytes from I/R-induced injury by controlling the *HAS2*/*FGF9* axis. These findings reveal the potential therapeutic significance of Tan IIA in alleviating CHD-related myocardial dysfunction.

## 1. Introduction

Coronary heart disease (CHD), also known as coronary artery disease (CAD), is characterized by atherosclerotic changes in the coronary arteries, resulting in impaired blood flow, which in turn leads to myocardial ischemia and hypoxia [1]. The incidence of CHD is mainly among people over 40 years old [2]. The incidence rate in men is higher than that in women, and it is more common in people engaged in mental work [3, 4]. The aging of the population and lifestyle changes are intimately linked to the increased prevalence of CHD [5]. Numerous clinical trials have verified the significance of several variables such as obesity, diabetes, hypertension, smoking, and high cholesterol in the development of CHD [6–8]. Additionally, an increased risk of CHD is associated with high levels of stress, physical inactivity, and alcohol consumption [9]. Research has demonstrated that CHD can result in long-term heart failure and acute myocardial infarction, both of which increase the risk of sudden cardiac death [10]. Research on coronary heart disease typically employs models to simulate CHD-related pathological processes, particularly ischemia/reperfusion (I/R) injury. I/R injury occur during the restoration of blood supply after a period of ischemia or hypoxia. While reperfusion is crucial for tissue survival, it also induces significant cellular damage. This paradoxical phenomenon exacerbates the initial ischemic injury through mechanisms such as oxidative stress, inflammation, calcium overload, and apoptosis. Through such approaches, researchers can delve into the cellular mechanisms of CHD and screen/test potential therapeutic drugs. Understanding and targeting specific molecular pathways involved in I/R injury are crucial for developing new therapeutic strategies to protect cardiac health and improve patient prognosis. The treatment of coronary artery disease is highly complex due to diverse causes, individual differences, and comorbidities. In recent years, advancements in precision medicine and personalized treatment strategies have significantly improved treatment outcomes. Emerging therapies, including biologics, gene therapy, and minimally invasive interventions [11], have further enhanced patient prognosis. Currently, medication therapy is the primary stay of therapeutic treatment for CHD. Compared with Western medicine, traditional Chinese medicine has the characteristics of multiple ingredients, multiple targets, and pleiotropic effects. Therefore, it is essential to have a complete understanding of the mechanisms behind CHD to delve into more effective traditional Chinese medicine remedies to promote disease prevention and management.

The vast arsenal of traditional Chinese medicine, which includes *Pueraria lobata*, Chuanqiong, *Salvia miltiorrhiza*, *Angelica sinensis*, and other remedies, has been the focus of more and more research on CHD in recent years. These remedies are known for their multitarget therapeutic concepts and holistic treatment methodologies. Given their ease of synthesis, transportation, storage, and management, small molecule compounds produced from herbal remedies are therefore essential to the development of drugs linked to CHD. A significant part of *Salvia miltiorrhiza*, salvianolic acid IIA (Tan IIA) demonstrates a range of pharmacological properties, including as anti-inflammatory, antioxidant, cardioprotective, immunomodulatory, and antitumor actions. It has been widely studied for its potential therapeutic benefits, particularly in cardiovascular diseases. Research by Xu H et al. demonstrated that Tan IIA regulates the proliferation and apoptosis of cardiac H9c2 cells through the miR-133a-3p/EGFR axis, thereby improving the progression of CAD, highlighting its potential as an intervention for CAD treatment [12]. Li W et al. found that Tan IIA inhibits oxidative low-density lipoprotein (ox-LDL)-induced vascular smooth muscle cell (VSMC) proliferation and migration by modulating the miR-137/TRPC3 axis, emphasizing its potential therapeutic role in alleviating atherosclerosis and cardiovascular diseases [13]. Tan IIA possesses strong anti-inflammatory properties. In the event of cardiomyocyte injury, inflammation is a critical factor that exacerbates tissue damage. By reducing the inflammatory response, Tan IIA can potentially mitigate the extent of the injury. The study of Xu et al. demonstrated that Puerarin-Tanshinone IIA (Pue-Tan) inhibits the formation of atherosclerotic inflammatory plaques and slows the progression of atherosclerosis by targeting the succinic acid/HIF-1*α*/IL-1*β* axis, lowering interleukin-1*β* (IL-1*β*) levels and interfering with succinic acid. Hypoxia-inducible factor 1*α* (HIF-1*α*) downregulation points to Pue-Tan's possible therapeutic benefit in vascular inflammation. Furthermore, Tan IIA reduces markers of oxidative stress, such as malondialdehyde (MDA) and increases the activity of antioxidant enzymes like superoxide dismutase (SOD). Therefore, current research has found that Tan IIA has many potential cardioprotective effects, such as reducing myocardial infarct size [14], improving cardiac function [15], lowering levels of proinflammatory cytokines, and reducing oxidative stress markers [16]. To completely comprehend the function of Tan IIA in CHD, further thorough study is necessary.

A member of the *fibroblast growth factor* (FGF) family, *fibroblast growth factor 9* (*FGF9*) is a protein-coding gene that is essential for controlling embryonic development, cell division, proliferation, and migration. According to the study of Yang F. et al., *FGF9* has a major role in boosting the proliferation of mouse spermatogonial stem cells (SSC), activates the p38 MAPK signaling pathway, and enhances the expression of the genes ETV5 and BCL6B [17]. Yusuf IO et al. found that *FGF9* induces neurite outgrowth in Huntington's disease (HD) striatal cells through the extracellular signal-regulated kinase (ERK) pathway [18]. Furthermore, *FGF9* initially activates ERK signaling, leading to upregulation of NF-kB, ultimately enhancing neurite outgrowth in HD striatal cells. For example, Ding W et al. pointed out that *FGF9*, as part of the antiapoptotic pathway involved in circLRP62-2 and hnRNPM, can enhance the survival of cardiomyocytes under hypoxic conditions and provide a potential therapeutic target for coronary heart disease and ischemic myocardial injury [19]. These results offer fresh perspectives on the molecular processes behind vascular regeneration following ischemia heart damage as well as the genetic foundation of coronary artery development.

A recent study has shown that *Hyaluronan Synthase 2* (*HAS2*) mutations may be involved in the pathogenesis of nonsyndromic ventricular septal defect (VSD) and play a key role in cardiac development [20]. In the pathophysiology of CHD, the precise role of *FGF9* and *HAS2* is not yet known. Tanshinone IIA plays a significant role in the treatment of cardiovascular diseases, but its mechanism of action on CHD remains unclear. Therefore, this study aimed to elucidate the effects of Tanshinone IIA on the viability and inflammation of cardiomyocytes subjected to I/R injury, and to explore whether Tanshinone IIA regulates I/R-induced cardiomyocyte injury through the modulation of the HAS2/FGF9 axis. This research provided a theoretical basis for the development of targeted interventions for CHD.

## 2. Materials and Methods

### 2.1. Analyzing in the GSE23561 Microarray Dataset for Differentially Expressed Genes (DEGs)

The CAD-related GSE23561 microarray dataset was obtained from the Gene Expression Omnibus (GEO; https://www.ncbi.nlm.nih.gov/gds/) database, with the help of GPL10775 platform, Human 50K Exonic Evidence-Based Oligonucleotide array. The dataset contains 35 samples, from which 6 CAD patient samples (as the case group) and 9 control group samples (as the control group) were selected. Differential gene expression analysis was performed on these 15 samples using the “Limma” package in R. To identify genes sensitive or crucial to experimental conditions, genes were selected based on the fold change (FC) criterion, and DEGs with FC > 2 were classified as upregulated and DEGs with FC < 0.5 were classified as downregulated. *p* value < 0.05 was set as the statistical significance level.

### 2.2. Analysis of Modules Related to GSE23561 Microarray Dataset

To elucidate complex gene interactions and delineate gene modules strongly associated with cardiovascular disease, we employed weighted gene co-expression network analysis (WGCNA) on the comprehensive GSE23561 microarray dataset. Specifically, gene expression data covering all genes in the GSE23561 microarray dataset (6 CAD patient samples and 9 control group samples) were converted into unscaled co-expression networks. Utilizing the “WGCNA” package in R, we ensured strict compliance with scale-free topology criteria, indicating biologically meaningful networks. Hierarchical clustering was applied to the adjacency matrix to identify modules of co-expressed genes. In this constructed framework, each module contains a set of co-expressed genes, distinguished by different colors for clarity and identification. For each module, the module eigengene (ME) was calculated. This ME represents the overall expression profile of the module. By performing module-shape analysis on these gene modules and clinical phenotypes (case and control samples), corresponding correlations and *p* values were calculated to identify modules that were significantly associated with CAD.

### 2.3. Construct of Protein-Protein Interaction (PPI) Network and Identify of Hub Genes

To delineate the PPI network among overlapping genes, we utilized the Search Tool for the Retrieval of Interacting Genes (STRING; https://string-db.org/) database. This comprehensive database facilitates the exploration of functional associations and interactions among proteins [21]. Subsequently, Cytoscape software was used to build the PPI network. To ensure the comprehensiveness and accuracy of target screening, we used the three techniques provided by the cytoHubba plug-in for Cytoscape to select the top 20 genes, which in turn allowed us to identify crucial nodes in the network: edge penetration component (EPC), maximum density of neighborhood components (DMNC), and maximum cluster centrality (MCC). These algorithms prioritize genes based on their relevance and centrality in the network, helping to discover hub genes related to CAD.

### 2.4. Bioinformatics Analysis Using the Sangerbox Platform

Sangerbox (https://vip.sangerbox.com/login.html) is an online bioinformatics analysis platform that provides interactive graphical analysis tools. In this study, we first used the Sangerbox platform to cross analyze the genes in the turquoise module and the DEGs in the GSE23561 microarray dataset for overlapping genes. Then, genes from three different algorithms of the overlapping genes PPI network were also cross-analyzed to finally identify the key overlapping genes. Finally, based on this platform, the Spearman correlation between the expression of the hub gene (*FGF9*) and *HAS2* was also analyzed for this study, with the significance threshold as *p* < 0.05.

### 2.5. Functional Enrichment Analysis of CAD-Related Genes

In order to perform functional enrichment analysis on the selected submodules, we employed the Database for Annotation, Visualization, and Integrated Discovery (DAVID; https://david.ncifcrf.gov/tools.jsp) database. This comprehensive analysis encompasses Kyoto Encyclopedia of Genes and Genomes (KEGG) pathway enrichment and Gene Ontology (GO) term enrichment [22, 23]. Molecular activities (MF), Cellular Components (CC), and Biological Processes (BP) are the three hierarchical categories into which GO categorization divides genes and protein activities. The results obtained are statistically significant when *p* < 0.05.

### 2.6. Cell Culture and Transfection

From the Chinese Academy of Sciences Cell Bank in Shanghai, China, human AC16 cardiac cells were collected. 10% fetal bovine serum (FBS) and 1% penicillin-streptomycin were added to DMEM during the cell culture process. Under usual conditions, the cell cultures were maintained at 37°C in an environment with 5% CO_2_. A *FGF9* expression vector or an empty vector (control) was used to transfect cells when they were 70-80% confluent. siRNA sequences 5′-GCTGCTTATATTGTTGGCTTT-3′ (first) and 5′-GGTTTGTGATTCAGACACT-3′ (second) were used at a concentration of 50 nM to knockdown HAS2 for different experiments. Lipofectamine 2000 was used for transfections, and cells were cultivated for 48 hours before further investigation.

### 2.7. *In Vitro* Simulation of I/R Injury in Human AC16 Cardiomyocytes and Intervention by Tan IIA

To imitate ischemia conditions *in vitro*, human AC16 cardiomyocytes were grown under either normoxia (21% O_2_) or hypoxia (1% O_2_). Firstly, cells were kept in a 37°C, 5% CO_2_ controlled incubator. Cells were treated with several doses of Tan IIA (0, 20, 40, 60 *μ*M) in a suitable cell culture medium. For the I/R injury model, a hypoxic atmosphere was established by flushing a hypoxic chamber with 1% oxygen, 5% carbon dioxide, and balanced nitrogen. Cells were first subjected to hypoxic conditions (1% O_2_) for 24 hours to generate ischemia, followed by 2 hours of reoxygenation to simulate reperfusion. The cells were returned to a normal incubator at 37°C with 5% CO_2_ during the reperfusion phase to restore normal oxygen levels. The control group cardiomyocytes were cultured under normal oxygen conditions in the CO_2_ incubator for the same duration.

### 2.8. RNA Isolation and Quantitative Real-Time Polymerase Chain Reaction (qRT-PCR)

TRIzol reagent (Thermo Fisher Scientific, USA) was used to extract total RNA from AC16 cells in accordance with the manufacturer's instructions. After that, the PrimeScript RT kit (Takara, Japan) was used to synthesize cDNA. A StepOnePlus Real-Time PCR System (Applied Biosystems, USA) was utilized for the qRT-PCR analysis, and SYBR Green PCR Master Mix (Applied Biosystems, USA) was utilized accordingly. Glyceraldehyde-3-phosphate dehydrogenase (GAPDH), the internal reference, was used to standardize the expression levels. The following primers were used in the amplification process: *FGF9*: Forward 5′-ATGGCTCCCTTAGGTGAAGTT-3′, Reverse 5′-CCCAGGTGGTCACTTAACAAAAC-3′; *HAS2* Forward 5′-CAGAATCCAAACAGACAGTTC-3′, Reverse 5′-TAAGGTGTTGTGTGTGACTG-3′; *PCNA*: Forward 5′-TCATTACACTAAGGGCCGAAGA-3′, Reverse 5′-GCACAGGAAAATTACAACAGCATC-3′; *Bax*: Forward 5′-CGGCGAATTGGAGATGAACTGG-3′, Reverse 5′-CTAGCAAAGTAGAAGAGGGCAACC-3′; *Caspase-3*: Forward 5′-CATGGAAGCGAATCAATGGACT-3′, Reverse 5′-CTGTACCAGACCGAGATGTCA-3′; *IL-1β*: Forward AGCTACGAATCTCCGACCAC, Reverse CGTTATCCCATGTGTCGAAGAA; *TNFα*: Forward CCTCTCTCTAATCAGCCCTCTG, Reverse GAGGACCTGGGAGTAGATGAG; *IL-6*: Forward CCTGAACCTTCCAAAGATGGC, Reverse TTCACCAGGCAAGTCTCCTCA; *GAPDH*: Forward 5′-GATGATTGGCATGGCTTT-3′, Reverse 5′-CACCTTCCGTTCCAGTTT-3′. The 2^−ΔΔCT^ technique was employed to assess the expression. GAPDH was the internal control.

### 2.9. Western Blot (WB) Assay

Protease and phosphatase inhibitors (Thermo Fisher Scientific, USA) were added to RIPA lysis solution (Thermo Fisher Scientific, USA) to facilitate the preparation of AC16 cell protein lysates. Next, the protein concentration in the lysates was measured using the BCA protein assay kit (Thermo Fisher Scientific, USA). For Western blotting (WB) analysis, equal amounts of protein were separated by SDS-PAGE and then deposited onto PVDF membranes (Millipore, USA). Primary antibodies against the following proteins were used to probe the membranes: *FGF9* (Thermo Fisher Scientific, Inc., 1 : 1,000); TNF-*α* (Abcam, 1 : 1,000); Cleaved Caspase-3 (Wanleibio Co., Ltd., 1 : 500); IL-6 (Abcam, 1 : 1,000); PCNA (Wanleibio Co., Ltd., 1 : 500); IL-1*β* (Abcam, 1 : 1,000); Bax (Cell Signaling Technology, 1 : 1,000); *HAS2* (Abgent Biotech, 1 : 100); GAPDH (Cell Signaling Technology, 1 : 5,000) as a loading control. The blots were identified following secondary antibody incubation, and ImageJ software was used to quantify the outcomes.

### 2.10. Assay for Cell Viability

The Cell Counting Kit-8 (CCK-8) assay (Dojindo, Japan) was used to measure cell viability. A CCK-8 solution was added to each well in a 96-well plate after the AC16 cells were seeded at a density of 5 × 10^3^ per well. Then the culture medium was replaced by DMEM medium alone and AC16 cells were treated with muscone. Cells were then constructed *in vitro* ischemia-reperfusion cell model as previous described. Cell viability was determined by measuring the absorbance at 450 nm using a microplate reader (Thermo Fisher Scientific, USA).

### 2.11. Flow Cytometry

Trypsin-EDTA (Gibco, USA) was used to separate AC16 cells for flow cytometric analysis, and PBS was used for washing. As directed by the manufacturer, the cells were stained using fluorescently labeled antibodies that were specific for SPDEF and S100A16 (Abcam, USA). A flow cytometer (BD Biosciences, USA) was used for the flow cytometry, and FlowJo software (FlowJo LLC, USA) was used to analyze the data.

### 2.12. Enzyme-Linked Immunosorbent Assay (ELISA)

With commercially available ELISA kits from Beyotime, the levels of TNF-*α*, IL-6, and IL-1*β* were measured in accordance with the manufacturer's instructions. With the use of a microplate reader, absorbance measurements were taken at 450 nm. By titrating the respective standards, standard curves were created. The BCA test was used to calculate the amount of cellular protein in each culture flask. The amounts of TNF-*α*, IL-1*β*, and IL-6 were converted to pg/*μ*g (cellular protein), normalized to the contents of cells, and then represented as a percentage of the controls. This approach ensured accurate comparisons across samples by accounting for variations in protein concentrations.

### 2.13. Determination of Superoxide Dismutase (SOD) Activity and Malondialdehyde (MDA) Levels

A commercially available superoxide dismutase activity test kit (Beyotime, China) was used to assess SOD activity in accordance with the manufacturer's instructions. In brief, AC16 cardiomyocytes were lysed, and the supernatant was collected postcentrifugation. The assay relies on the ability of SOD to catalyze the dismutation of superoxide anions, with the rate of reduction of a tetrazolium salt serving as an indirect measure of enzyme activity. At 450 nm, absorbance was measured, and the Bradford technique was used to express SOD activity in relation to protein content. Lipid peroxidation (MDA) test kit (Beyotime, China) was used to measure MDA levels, in accordance with the manufacturer's instructions. Following the extraction and homogenization of the cells, thiobarbituric acid (TBA) and MDA in the sample interacted at a high temperature and in an acidic environment to generate a TBA-MDA adduct. The adduct was quantified spectrophotometrically by measuring the absorbance at 532 nm. In comparison to the control group, the results were displayed after being normalized based on the total protein content.

### 2.14. Statistical Analysis

To scrutinize our datasets, we employed the R programming language along with the R packages “Limma” and “WGCNA.” We used SPSS software for statistical analysis, and GraphPad Prism software was used to obtain images. All data are provided as mean ± SD, and Student's *t*-test was used for statistical analysis to assess intergroup differences. Analysis of variance (ANOVA) and Tukey's post hoc test were used to determine differences among several groups. For statistical significance, a cutoff of *p* < 0.05 was established.

## 3. Results

### 3.1. Identification of Gene Modules Associated with Clinical Characteristics of CAD

**Figure 1:**
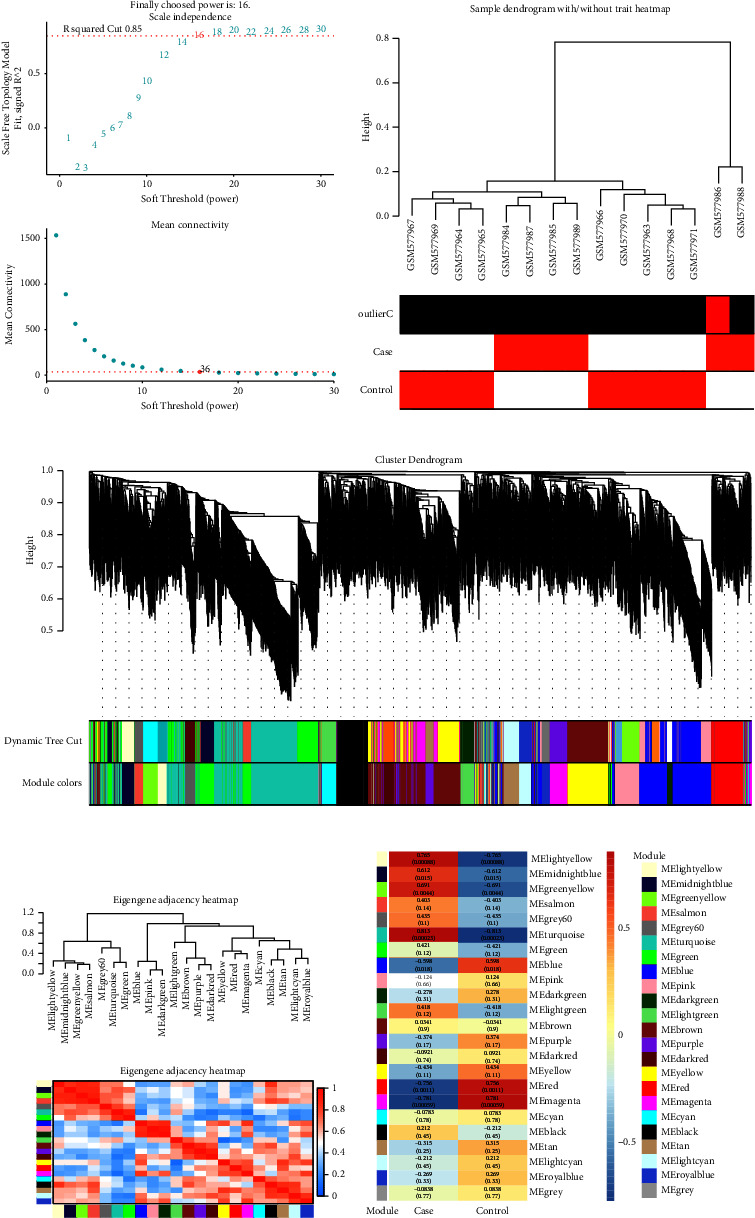
Gene co-expression network analysis of Peng Yu microarray dataset. (a) Network topology analysis for different soft threshold powers. The upper panel shows the scale-free fit index (*y*-axis) as a function of soft threshold power (*x*-axis), and the lower panel shows mean connectivity (*y*-axis) for different soft thresholds (x weeks). (b) The top dendrogram shows sample clustering of gene expression data, with different branches representing different samples. (c) Dendrogram showing clusters of co-expressed genes, with different gene modules represented by different colors. (d) Heatmap of module eigengene adjacencies. Each cell contains the corresponding correlation between the module signature gene pairs, with colors ranging from blue (low adjacency) to red (high adjacency). (e) Heat map of gene module-clinical trait relationships, with the color scale on the right indicating correlations from high (red) to low (blue).

It was found that 16 was the optimal soft threshold power for fitting the scale-free topology model, as shown in Figure 1(a). Following the establishment of this soft threshold power, we proceeded with the analysis of 15 samples from the GSE23561 dataset, as depicted in Figure 1(b). Utilizing the WGCNA method, genes were classified into different modules based on co-expression patterns among samples; each module was assigned a distinct color (Figure 1(c)). To unravel the interrelations among the identified modules, a meticulous examination of the adjacency of feature genes was performed (Figure 1(d)). Among the various modules, the turquoise module exhibited a significant correlation coefficient of 0.813 with the samples, indicative of a pronounced relationship (Figure 1(e)). This noteworthy correlation underscores the potential biological relevance of genes within the turquoise module to the GSE23561 microarray dataset.

### 3.2. Integrated Analysis Reveals Key Overlapping Genes and Enriched Pathways in CAD Using GSE23561 Microarray Dataset

**Figure 2:**
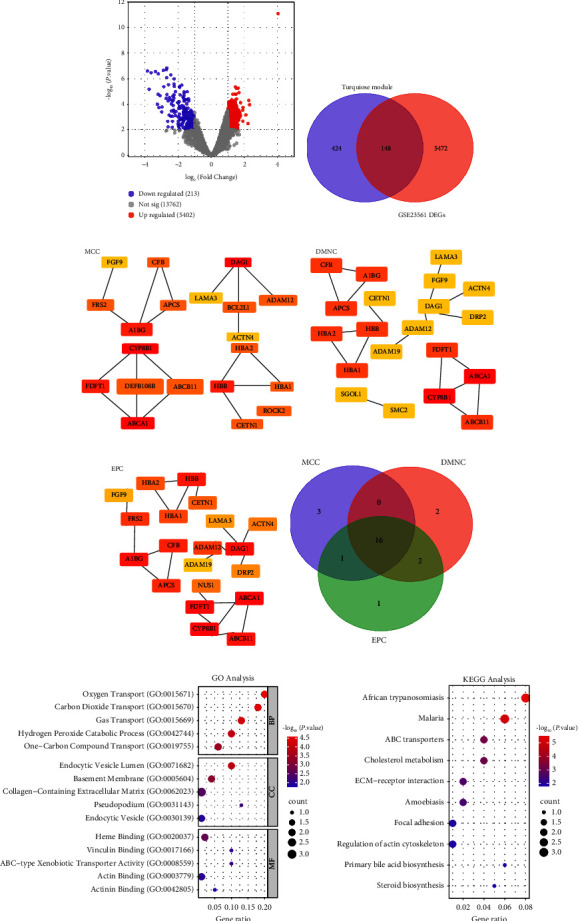
Comprehensive analysis of CAD-related genes in the GSE23561 microarray dataset. (a) Volcano plot displaying identified DEGs from the GSE23561 microarray dataset. The *X*-axis (log_2_ fold change) represents the log_2_ fold change in gene expression between CAD and control samples. The *Y*-axis (-log_10_*p* value) indicates the statistical significance of differential expression. Higher values represent more significant results. Each point represents an individual gene. Upregulated DEGs are depicted in red, and downregulated DEGs are depicted in blue. (b) Venn diagram illustrating the overlap of turquoise module genes with DEGs. (c) PPI network module derived from the MCC algorithm, displaying 20 nodes and 17 edges. (d) PPI network module generated using the DMNC algorithm, consisting of 20 nodes and 18 edges. (e) PPI network module constructed based on the EPC algorithm, with 20 nodes and 20 edges. (f) Sixteen key overlapping genes are shown in a Venn diagram showing the intersection of genes found in three PPI network modules. (g) GO enrichment analysis of the function of overlapping genes based on BP, CC, and MF. The abscissa is GeneRatio, and the ordinate is an enrichment term. The larger the dots, the more genes are enriched. (h) KEGG enrichment analysis predicted the pathways of overlapping gene involvement. DEGs: differential expressed genes, GO: gene ontology, BP: biological process, CC: cell component, MF: molecular function. KEGG: Kyoto Encyclopedia of Genes and Genomes.

Differential expression analysis of CAD patients and the control group within the GSE23561 dataset revealed 5407 upregulated DEGs and 213 downregulated DEGs (Figure 2(a)). Subsequent analysis emphasized an intersection of 148 genes between the DEGs in the GSE23561 dataset and the turquoise module containing 572 genes (Figure 2(b)). PPI networks analysis on these 148 overlapping genes identified three key gene modules based on MCC (20 nodes and 17 edges), DMNC (20 nodes and 18 edges), and EPC (20 nodes and 20 edges) (Figures 2(c), 2(d), and 2(e)). As shown in Figure 2(f), 16 key overlapping genes were obtained from these gene modules. Functional enrichment analysis of these genes revealed significant enrichment in GO terms. In BP, terms such as Oxygen Transport, Carbon Dioxide Transport, and Hydrogen Peroxide Catabolic Process exhibited notable enrichment. In CC, enrichment was observed in terms like Endocytic Vesicle, Basement Membrane, and Pseudopodium. MF terms, including Heme Binding, Vinculin Binding, and Actin Binding, demonstrated significant enrichment (Figure 2(g)). Furthermore, KEGG pathway analysis indicated significant enrichment in pathways such as Steroid Biosynthesis, Focal Adhesion, and Regulation of Actin Cytoskeleton (Figure 2(h)). More investigation of the expression levels of the 16 key overlapping genes in the Control and CAD groups within the GSE23561 dataset revealed *FGF9* to exhibit more significant differential expression, thus chosen as the central gene for further investigation (Supplementary Table 1).

### 3.3. Tan IIA Increases AC16 Cell Viability, Reduces Apoptosis, and Oxidative Stress Levels under Hypoxic Conditions

**Figure 3:**
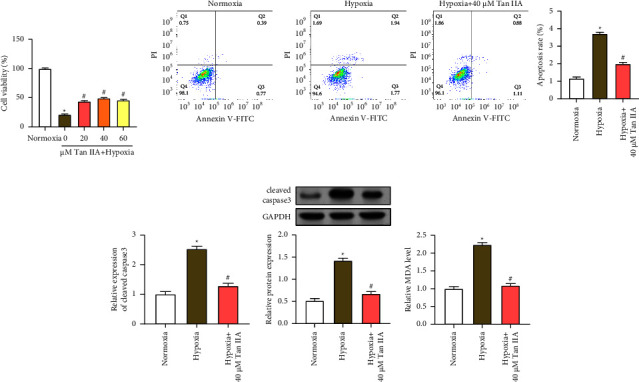
Effects of Tan IIA on cardiomyocyte growth viability, apoptosis, and lipid peroxidation under hypoxic conditions. (a) CCK-8 detects the cell viability of AC16 cardiomyocytes under normoxia conditions and after hypoxic treatment with or without different concentrations (0, 20, 40, 60 *μ*M) of Tan IIA. (b) Flow cytometric analysis of AC16 cardiomyocytes stained with Annexin V-FITC/PI under normoxic conditions, hypoxia, and 40 *μ*M Tan IIA-treated hypoxia. The right panel quantifies the percentage of apoptotic cells. (c and d) qRT-PCR and WB analysis showed the relative expression levels of cleaved caspase-3 mRNA in AC16 cardiomyocytes under normoxic conditions, hypoxia, and 40 *μ*M Tan IIA-treated hypoxic conditions. (e) Relative levels of MDA in AC16 cardiomyocytes under normal oxygen condition, hypoxia condition, and 40 *μ*M Tan IIA hypoxia condition. ⁣^∗^*p* < 0.05 vs. normoxia group, ^#^*p* < 0.05 vs. hypoxia group.

CCK-8 was used to measure the viability of AC16 cells in both normoxic and hypoxic settings following treatment with varying doses (0, 20, 40, and 60 *μ*M) of Tan IIA. The findings demonstrated that, in comparison to normoxic settings, hypoxia dramatically decreased the viability of AC16 cardiomyocytes. Pretreatment with Tan IIA showed a dose-dependent protective effect against hypoxia-induced cytotoxicity, with 20, 40, and 60 *μ*M concentrations of Tan IIA significantly increasing cell viability compared to untreated hypoxic cells. Notably, the rescue effect on cell viability was most pronounced at the 40 *μ*M Tan IIA concentration chosen in subsequent experiments (Figure 3(a)). Subsequently, the apoptosis of AC16 cardiomyocytes under normoxic and hypoxic circumstances, as well as under combination treatment of hypoxia and 40 *μ*M Tan IIA, was assessed by flow cytometry. It was found that under hypoxia conditions, the cell apoptosis rate increased. However, after adding 40 *μ*M Tan IIA, the cell apoptosis rate decreased to a certain extent (Figure 3(b)). qRT-PCR and WB detected that Cleaved Caspase-3 levels were significantly increased under hypoxia conditions compared with normoxia conditions, and the addition of 40 *μ*M Tan IIA could effectively inhibit this increase (Figures 3(c) and 3(d)). Finally, the lipid peroxidation MDA assay was used to evaluate the extent of lipid peroxidation in AC16 cells. Lipid peroxidation in the hypoxic group was found to be considerably higher than in the normoxic control group, as determined by assessing the expression level of MDA. However, the addition of 40 *μ*M Tan IIA reduced the level of MDA and effectively inhibited the hypoxia-induced increase in lipid peroxidation levels (Figure 3(e)).

### 3.4. Tan IIA Reduces Cardiomyocyte Injury Induced by I/R Stimulation

**Figure 4:**
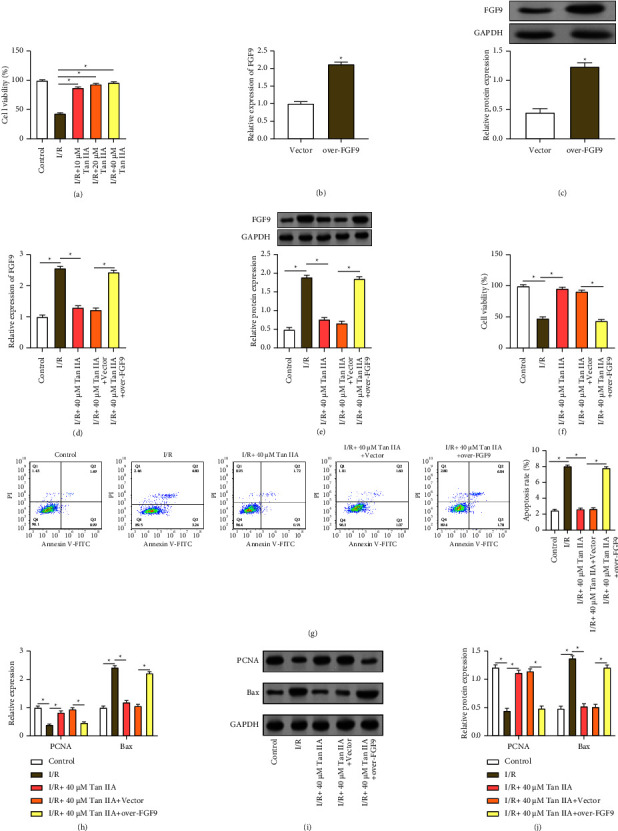
Tan IIA attenuates I/R-induced AC16 cardiomyocyte injury by regulating *FGF9*. (a) CCK-8 activity assay. Assessment of AC16 cardiomyocyte proliferation under I/R treatment and Tan IIA intervention. (b and c) *FGF9* overexpression efficiency. Validation of *FGF9* overexpression in AC16 cardiomyocytes by qRT-PCR and Western blotting. (d and e) Expression of *FGF9* under different conditions. The expression of *FGF9* under I/R treatment, Tan IIA intervention and *FGF9* co-overexpression was detected by qRT-PCR and Western blotting. (f and g) Cell viability and apoptosis analysis. CCK-8 and flow cytometry assessed AC16 cardiomyocytes under various conditions (I/R, I/R+ 40 *μ*M Tan IIA, I/R+ 40 *μ*M Tan IIA + vector, I/R+ 40 *μ*M Tan IIA + over-*FGF9*) cell viability and apoptosis rate. (h–j) PCNA and Bax expression level. PCNA and Bax expression levels under different conditions (I/R, I/R+ 40 *μ*M Tan IIA, I/R+ 40 *μ*M Tan IIA + vector, I/R+ 40 *μ*M Tan IIA + over-*FGF9*) were examined by qRT-PCR and Western blotting. ⁣^∗^*p* < 0.05.

Given the close association between myocardial I/R injury and CHD, our goal was to validate the simulation of myocardial ischemic conditions by ischemia-reperfusion therapy. The CCK-8 results indicated that cell viability decreased following I/R treatment, but it rose in a dose-dependent way with the addition of various doses of Tan IIA (Figure 4(a)). Subsequently, *FGF9* overexpression plasmid was transfected into AC16 cells, and qRT-PCR was used to determine the effective overexpression efficiency (Figures 4(b) and 4(c)). Further studies found that *FGF9* expression was markedly elevated in AC16 cells after I/R treatment. Adding Tan IIA seemed to attenuate this increase, but this attenuation was restored after being combined with overexpressed *FGF9* (Figure 4(d)). The results of protein level analysis also showed a similar trend (Figure 4(e)), suggesting that Tan IIA may be able to reduce the increase of *FGF9* expression brought on by I/R. Cell viability and apoptosis under I/R treatment, I/R treatment combined with 40 *μ*M Tan IIA, and *FGF9* co-overexpression were then assessed using CCK-8 and flow cytometry. From the results in Figures 4(f) and 4(g), the I/R treatment resulted in decreased cell viability and increased apoptosis, whereas the addition of 40 *μ*M Tan IIA promoted cell proliferation and inhibited apoptosis, reversing the damage of I/R on AC16 cells. Notably, these reversal effects were restored upon co-overexpression of *FGF9*. In addition, qRT-PCR and WB experiments showed that adding 40 *μ*M Tan IIA could rescue the decrease in the expression of cell cyclin (PCNA) and increase in the expression of apoptotic protein (Bax) caused by I/R treatment. Importantly, co-overexpression of *FGF9* again resulted in decreased PCNA expression and increased BAX expression at levels similar to those after I/R injury (Figures 4(h), 4(i), and 4(j)). In summary, Tan IIA treatment ameliorates ischemia-reperfusion-induced AC16 cell injury, providing potential effects on CHD.

### 3.5. Tan IIA Protects AC16 Cells from I/R-Induced Injury by Upregulating *FGF9* Expression

**Figure 5:**
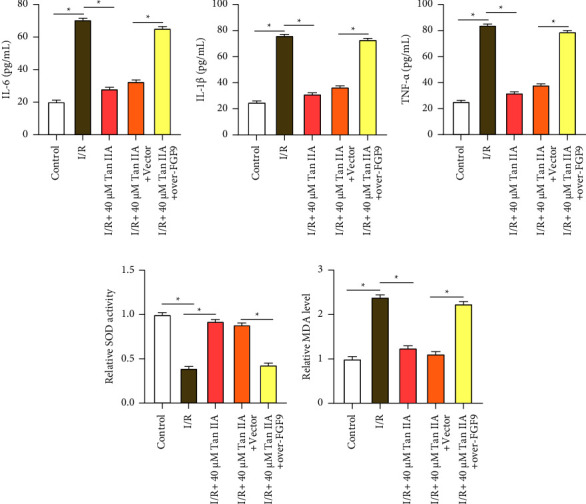
Tan IIA inhibits inflammation and oxidative stress in I/R-induced AC16 cardiomyocytes by regulating *FGF9*. (a–c) ELISA experiments evaluate the response to Tan IIA, I/R treatment, and *FGF9* co-overexpression on IL-6 (a), IL-1*β* (b), and TNF-*α* (c) secretion. (d) Superoxide dismutase activity assay for SOD activity after I/R treatment, I/R treatment + 40 *μ*M Tan IIA and co-overexpression of *FGF9*. (e) Lipid peroxidation assay to detect MDA levels after I/R treatment, I/R treatment + 40 *μ*M Tan IIA, and co-overexpression of *FGF9*. SOD: superoxide dismutase; MDA: malondialdehyde. ⁣^∗^*p* < 0.05.

The ELISA experiment revealed that proinflammatory cytokines (IL-6, IL-1*β*, and TNF*α*) were considerably elevated in AC16 cells following I/R-induced cell injury. In contrast to the I/R group, treatment with 40 *μ*M Tan IIA could reduce levels of these cytokines. Interestingly, combined treatment with Tan IIA and overexpressed *FGF9* under I/R induction resulted in cytokine levels comparable to those in the control group, suggesting a synergistic anti-inflammatory effect (Figures 5(a), 5(b), and 5(c)). Furthermore, I/R injury resulted in reduced SOD activity, which was substantially enhanced by Tan IIA treatment; however, the introduction of *FGF9* overexpression attenuated these effects (Figure 5(d)). A hallmark of lipid peroxidation MDA levels increased in the I/R group. Tan IIA treatment reduced MDA levels, but this decrease was reversed in I/R induction, combined treatment with Tan IIA, and overexpressed *FGF9* (Figure 5(e)).

### 3.6. Regulation of *FGF9* by *HAS2* Knockdown Attenuates I/R-Induced AC16 Cell Injury

**Figure 6:**
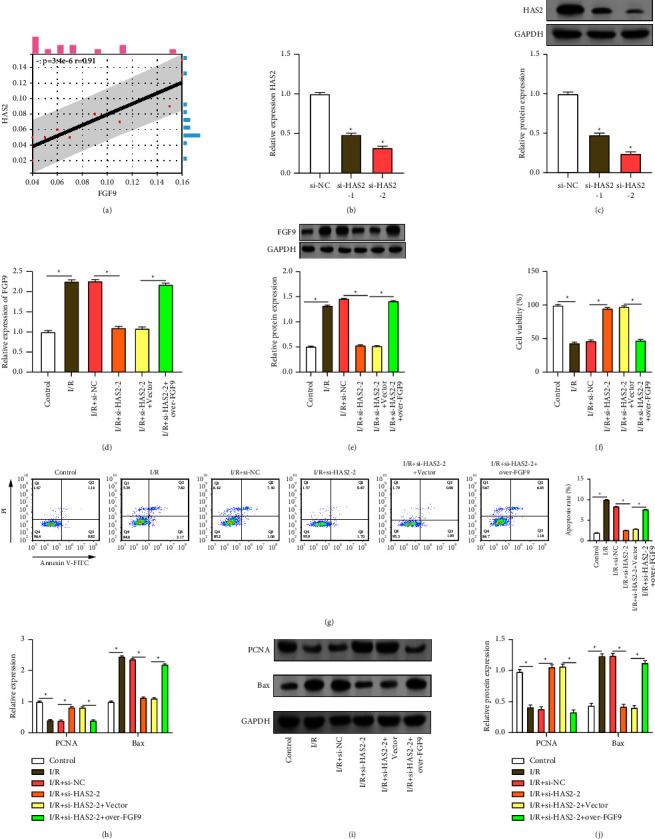
Regulation of AC16 cell viability and apoptosis after I/R injury by *HAS2* downregulation and *FGF9* overexpression. (a) Scatter plot illustrating the positive correlation and interaction between the *FGF9* gene and *HAS2*. (b-c) Confirmation of *HAS2* knockdown efficiency in AC16 cardiomyocytes by qRT-PCR and Western blotting. (d-e) Expression of *FGF9* under different conditions. The expression of *FGF9* under I/R treatment, *HAS2* knockdown and *FGF9* co-overexpression was detected by qRT-PCR and Western blotting. (f and g) Viability and apoptosis of AC16 cardiomyocytes under different conditions (Control, I/R, I/R + si-NC, I/R + si-*HAS2*-2, I/R + si-*HAS2*-2 + vector, I/R + si-*HAS2*-2 + over-*FGF9*) were detected by CCK-8 and flow cytometry. (h–j) PCNA and Bax expression levels under different conditions (control, I/R, I/R + si-NC, I/R + si-*HAS2*-2, I/R + si-*HAS2*-2 + vector, I/R + si-*HAS2*-2 + over-*FGF9*) were examined by qRT-PCR and Western blotting.

**Figure 7:**
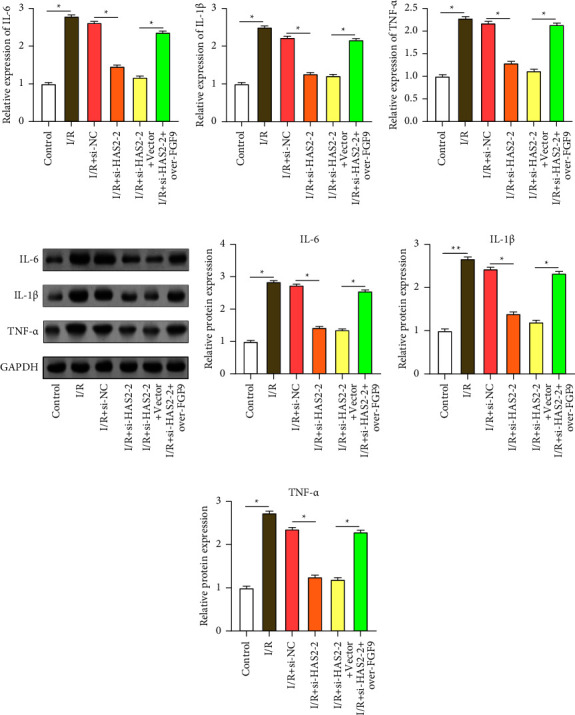
*HAS2* knockdown reduces I/R-induced inflammation in AC16 cardiomyocytes. (a–g) qRT-PCR (a–c) and Western blotting (d-e) analysis of the expression levels of IL-6, IL-1*β*, and TNF-*α* in AC16 cells under different treatment conditions (control, I/R, I/R + si-NC, I/R + si-*HAS2*-2, I/R + si-*HAS2*-2 + vector, I/R + si-*HAS2*-2 + over-*FGF9*). ⁣^∗^*p* < 0.05.

Analysis on the Sangerbox platform revealed a positive correlation and interaction between *FGF9* and *HAS2*, as depicted in the scatter plot (Figure 6(a)). AC16 cells were transfected with two specific siRNA sequences targeting *HAS2*. qRT-PCR and WB analysis showed that compared with si-NC, both si-*HAS2*-1 and si-*HAS2*-2 significantly reduced *HAS2* mRNA and protein levels, among which the reduction of si-*HAS2*-2 was more obvious (Figures 6(b) and 6(c)). Further investigation into the impact of *HAS2* knockdown on *FGF9* expression revealed that the introduction of *HAS2* knockdown plasmid weakened the I/R-induced elevation of *FGF9* expression. Moreover, this weakening effect was reversed upon co-transfection with the *FGF9* overexpression plasmid (Figures 6(d) and 6(e)). CCK-8 (Figure 6(f)) and flow cytometry (Figure 6(g)) analysis revealed that an important reduction in cell viability and a considerable rise in the rate of cell apoptosis following I/R induction. After I/R injury, *HAS2* knockdown results in a decrease in the rate of cell apoptosis and an increase in cell viability. This implies that *HAS2* knockdown may protect against cell damage brought on by I/R. Notably, co-treatment with overexpression of *FGF9* resulted in decreased cell viability and increased apoptosis, inhibiting the protective effect of knockdown *HAS2* on cells. Further investigation of PCNA and Bax expression revealed that *HAS2* knockdown could reverse I/R-induced decrease of PCNA expression and increase Bax expression. However, co-transfection with the *FGF9* overexpression plasmid reversed these effects (Figures 6(h), 6(i), 6(j)). Finally, qRT-PCR and Western blot analyses of inflammatory factors (TNF-*α*, IL-1*β*, and IL-6) secretion showed that compared to I/R, the knockout of *HAS2* inhibited the increase in TNF-*α*, IL-1*β*, and IL-6, thereby suppressing the I/R-induced inflammatory response. However, transfection with the *FGF9* overexpression plasmid reversed the protective effect of *HAS2* knockout on AC16 (Figures 7(a), 7(b), 7(c), 7(d), 7(e), 7(f),and 7(g)). These results suggest that *HAS2* is essential for modulating the cellular response to I/R injury and that knockdown of *HAS2* reduces apoptosis and improves cell viability, which may be achieved by regulating *FGF9* expression and inflammatory cytokine production.

### 3.7. Tan IIA Inhibits *HAS2*/*FGF9* Axis Expression, Mitigating I/R-Induced AC16 Cell Injury

**Figure 8:**
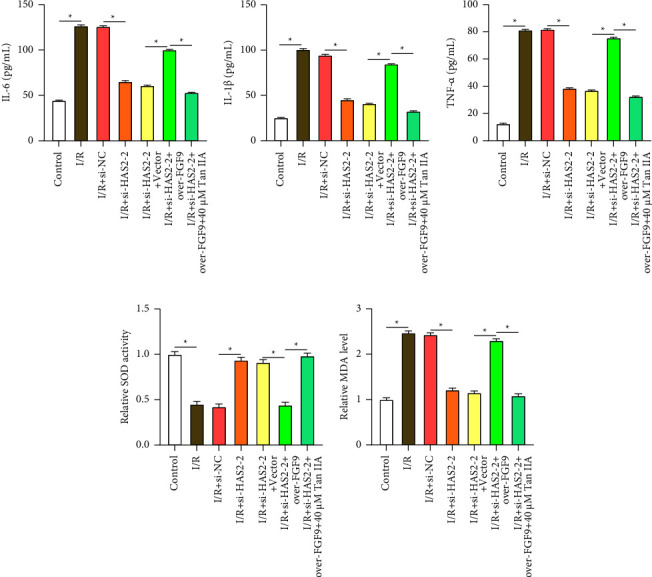
Tan IIA inhibits the *HAS2*/*FGF9* axis and alleviates I/R-induced AC16 cell injury. (a–c) ELISA experiments evaluating secretion pair levels of IL-6 (a), IL-1*β* (b), and TNF-*α* (c) in different groups (control, I/R, I/R + si-NC, I/R + si-*HAS2*-2, I/R + si-*HAS2*-2 + vector, I/R + si-*HAS2*-2 + over-*FGF9*, I/R + si-*HAS2*-2 + over-*FGF9* + 40 *μ*M Tan IIA). (d) Detection of SOD activity in AC16 cardiomyocytes under different treatment conditions (control, I/R, I/R + si-NC, I/R + si-*HAS2*-2, I/R + si-*HAS2*-2 + vector, I/R + si-*HAS2*-2 + over-*FGF9*, I/R + si-*HAS2*-2 + over-*FGF9* + 40 *μ*M Tan IIA). (e) Lipid peroxidation MDA assay. Detection of MDA levels in AC16 cardiomyocytes under different treatment conditions (control, I/R, I/R + si-NC, I/R + si-*HAS2*-2, I/R + si-*HAS2*-2 + vector, I/R + si-*HAS2*-2 + over-*FGF9*, I/R + si-*HAS2*-2 + over-*FGF9* + 40 *μ*M Tan IIA). SOD: superoxide dismutase; MDA: malondialdehyde. ⁣^∗^*p* < 0.05.

In AC16 cells, I/R injury significantly elevated inflammatory cytokines and oxidative stress. *HAS2* knockdown attenuated the I/R-induced elevation of TNF-*α*, IL-6, MDA, and IL-1*β* levels as well as the decrease in SOD activity, suggesting a protective effect against I/R-induced cellular injury. However, transfection with *FGF9* overexpression plasmid attenuated these effects. Remarkably, addition of 40 *μ*M Tan IIA inhibited the increase of proinflammatory cytokines and MDA and increased SOD activity, suggesting a potential protective effect of Tan IIA against I/R-induced proinflammatory and oxidative effects (Figures 8(a), 8(b), 8(c), 8(d), and 8(e)). These findings suggest that by inhibiting the expression of the *HAS2/FGF9* axis, Tan IIA can lessen the damage that I/R causes to AC16 cells.

## 4. Discussion

In the realm of CHD, the coronary arteries play a pivotal role as the primary vessels supplying blood to the heart. Obstructions or constrictions in these arteries can impede the adequate delivery of blood and oxygen to the cardiac muscles, culminating in the manifestation of CHD [24]. We utilized the WGCNA method to partition the co-expression genes among samples in the GSE23561 dataset into different modules, selecting the turquoise module with a high sample correlation coefficient (0.813). After our enrichment analysis of the genes in GSE23561 that overlapped, the results showed several enriched terms, including oxygen transport, steroid biosynthesis, regulation of actin cytoskeleton, etc. Oxygen transport is a basic biological process that is crucial to cardiac function. Insufficient oxygen transport can lead to ischemia, myocardial hypoxia, and cell damage or death. Therefore, our enrichment results indicate that overlapping genes may participate in the pathogenesis of CHD. Insights from the research conducted by Wang S et al. shed light on the significant role of the *FGF9* subfamily, encompassing *FGF9*, 16, and 20, in cardiac development, postnatal cardiac homeostasis maintenance, and the facilitation of functional adaptation and survival in adult myocardium [25]. Notably, their findings underscored the potential of these subfamily members as therapeutic targets for cardiac protection and vascular reconstruction, particularly in the aftermath of myocardial infarction. Further contributions to our understanding come from Sun J et al., who revealed the regulatory impact of lncRNA FAF in suppressing cardiac fibrosis induced by angiotensin II in cardiac fibroblasts [26]. This inhibitory effect was mediated through the targeting of *FGF9* and modulation of the TGF*β*1-P-Smad2/3 signaling pathway, emphasizing its prospective therapeutic role in preventing cardiac fibrosis. The study of Said SS et al. introduced a novel avenue by controlling the delivery of *FGF9* from biodegradable poly (ester amide) fibers, demonstrating potential applications in therapeutic angiogenesis [27]. This approach holds promise in addressing ischemic vascular diseases, including coronary artery diseases, by promoting the regeneration of stable and functional new vascular systems. Moreover, the investigation of Ng A et al. into the association of phosphatidylinositol glycan anchor biosynthesis class 3 (Gpc3) deficiency with congenital heart defects highlighted *FGF9* as a potential factor in the molecular mechanisms underlying coronary artery development [28]. This insight suggests a correlation between *FGF9* and the genetic basis of understanding coronary artery diseases. In alignment with these perspectives, the present study employed WGCNA to analyze patients with coronary artery disease and control groups. Through iterative screening of overlapping genes, the study identified *FGF9* as a hub gene with more pronounced expression, contributing to the elucidation of its significant role in the context of CHD.

In the current landscape of CHD therapeutics, aspirin, statins, and antihypertensive medications stand as the primary pharmaceutical interventions [29]. However, the imperative remains to develop CHD treatment modalities that are not only cost-effective but also safe and efficacious. Tan IIA has been employed in China for the treatment of CHD for many years, with numerous studies elucidating its effects on myocardial cells [30]. Studies by Xu et al. demonstrated that Tan IIA alleviates myocardial cell damage induced by oxygen-glucose deprivation by inhibiting NF-*κ*B activity and upregulating the expression of the long noncoding RNA AK003290, thereby reducing inflammation [31]. Deng et al. discovered that Tan IIA prevents acute ethanol-induced myocardial cell apoptosis by inhibiting *PDCD4* expression and activating the PI3K/Akt pathway, presenting a potential therapeutic strategy for ethanol-induced cardiac injury [32]. Ma et al. found that Tan IIA, a key component of Danshen, mitigates atherosclerosis and endothelial inflammation in CAD by inhibiting COX-2 expression, offering a potential therapeutic avenue [33]. Moreover, the research of Ren ZH et al. on Tan IIA revealed its potential cardiac protective effect by attenuating the inflammatory response in myocardial infarction rats through the reduction of MCP-1 expression [34]. In our experiments, the promotion of AC16 cell viability increased with higher doses of Tan IIA, reaching the best effect at the optimal dose of 40 *μ*M. This indicates that Tan IIA can enhance the survival rate of cardiac cells under hypoxic conditions and exhibits a dose-dependent protective effect on the heart. Additionally, studies have shown that under hypoxic conditions, Tan IIA can promote the expression of the cell cyclin protein PCNA and inhibit apoptosis and the expression of the apoptotic protein Bax. This is consistent with the findings of Xu et al. [35], who discovered that Tan IIA inhibits apoptosis in cardiomyocytes. Furthermore, Tan IIA could also reduce oxidative stress levels in a hypoxic environment. Based on studies of the functions of *FGF9* and Tan IIA in I/R-induced injury, overexpression of *FGF9* inhibits cell viability and promotes cell apoptosis, while Tan IIA has the ability to alleviate I/R-induced damage in AC16 cells. It is noteworthy that Tan IIA protected AC16 cells from I/R-induced damage by downregulating the production of *FGF9*.

The protein-encoding gene *HAS2* is linked to several metabolic and glycosaminoglycan metabolism pathways. The findings of the study by Petz et al. demonstrated the critical function of *HAS2* in the healing process that occurs after an ischemia-reperfusion injury [36]. This function is achieved by enhancing the survival of macrophages and the myofibroblast response. Lagendijk AK et al. identified the crucial involvement of *HAS2* in heart valve formation, regulated by microRNA-23 (miR-23) inhibition, which limits endocardial cushion cell differentiation by modulating the production of extracellular hyaluronic acid [37]. Azizidoost et al. demonstrated that inhibiting microRNA-23 (miR-23) prevents myocardial ischemia/reperfusion injury (MIRI) by upregulating acetyl hyaluronan synthase 2 (*Has2*) expression, promoting bone marrow mesenchymal stem cell (BMSC) differentiation into cardiomyocytes, possibly through Wnt pathway activation [38]. Sun et al. discovered that *FGF9* facilitates the expression of palatal *HAS2* via the Wnt/*β*-catenin/TCF7L2 pathway, where TCF7L2 activates palatal *HAS2* transcription [39]. Existing studies have shown a positive correlation between the expression of *HAS2* and *FGF9* [39]. We determined the positive correlation and interaction between *HAS2* and *FGF9Z* by Sangerbox platform analysis. Our experiment delved into the intricate relationship between *HAS2* and *FGF9*, revealing that knocking down *HAS2* could ameliorate I/R-induced AC16 cell injury by mitigating the decrease in cell viability, increase in apoptosis, and inflammatory response caused by *FGF9* overexpression. Subsequent experiments unveiled that Tan IIA, in turn, could also inhibit the expression of the *HAS2/FGF9* axis, enhancing cell viability and SOD activity, and suppressing the increases in apoptosis, proinflammatory cytokines, and MDA. This provided a promising approach for improving I/R-induced damage in AC16 cells. The aforementioned discoveries enhance our comprehension of the complex relationship between *HAS2* and *FGF9* and highlight the possible therapeutic consequences of Tan IIA when it comes to cardiac I/R damage.

In this experiment, we further investigated the link between myocardial I/R injury and coronary heart disease by establishing a model simulating such injury. Postmyocardial ischemia, the restoration of blood supply (reperfusion) is critical for recovering myocardial function. During reperfusion, the reintroduction of oxygen can trigger inflammatory responses and the generation of oxygen-free radicals, processes that may inflict damage upon myocardial cells [40, 41]. Inflammation not only is essential to the occurrence and progression of atherosclerosis but is also pivotal in the stability of established atherosclerotic plaques [42, 43]. Studies have shown that elevated levels of TNF-*α*, IL-1*β*, and IL-6 are frequently associated with the development of myocardial injury [44]. These elevated TNF-*α*, IL-1*β*, and IL-6 levels might aggravate cardiac damage by causing inflammation, which in turn can result in cardiomyocyte mortality [45]. While elevated levels of MDA suggest higher lipid peroxidation and cell membrane damage, SOD functions as a preventive enzyme to decrease oxidative stress by proportionating superoxide radicals. In our study, leads to reduced cell viability and increased apoptosis, levels of proinflammatory cytokines, and oxidative stress, manifesting as increased MDA levels and decreased SOD activity, in AC16 cells. By reducing oxidative stress markers and inflammation, *HAS2* knockdown mitigated these effects. Interestingly, overexpression of *FGF9* can diminish the protective effects of *HAS2* knockdown, but the addition of Tan IIA downregulates *FGF9* expression and restores the protective effects. Therefore, the combined use of *FGF9* and Tan IIA may produce a dual effect: on one hand, Tan IIA can attenuate the adverse effects of *FGF9* overexpression on cells, and on the other hand, Tan IIA can enhance its own protective effects by lowering *FGF9* levels. This synergistic effect may lead to more effective cardiac protection, reducing the severity of I/R injury. This suggests that Tan IIA may improve cell I/R injury by inhibiting the *HAS2*/*FGF9* axis. This highlights the potential of Tan IIA as a therapeutic agent to mitigate the detrimental effects of I/R injury. Through the assessment of inflammatory factors, oxidative stress markers, and free radical response under various circumstances, our goal is to gain more insight into the intricate interactions among Tan IIA, *HAS2*, and *FGF9* in controlling oxidative stress levels, potentially illuminating their functions in CHD and myocardial I/R injury.

Certainly, this study also has some limitations. This study could be further strengthened methodologically by integrating other holographic disciplines (e.g., metabolomics, spatial transcriptomics, ATAC-seq, or proteomics) to increase its persuasiveness. Additionally, we focused on the *HAS2*/*FGF9* axis, other signaling pathways and molecular mechanisms might also play significant roles in the cardioprotective effects of Tan IIA. This study may not have captured the complete network of interactions involved. A further limitation is that this study was conducted using AC16 cells *in vitro*, which cannot fully replicate the complex conditions within the human body. Therefore, further validation in animal models and clinical studies is needed to confirm the relevance and applicability of our findings in more complex biological systems. Our next step is to plan studies in animal models of cardiac I/R injury. These studies should include assessments of cardiac function, histopathological examinations, and measurements of systemic inflammatory and oxidative stress markers. We will also utilize publicly available single-cell sequencing data on coronary artery disease to validate and supplement key gene loci [46, 47], while simultaneously exploring other potential molecular mechanisms and signaling pathways through which Tan IIA may impact cardiac cells.

## 5. Conclusion

This study elucidates the cardioprotective properties of Tan IIA under I/R injury conditions. *HAS2* knockdown can alleviate I/R-induced cell damage, reduce cell apoptosis, and enhance cell viability by modulating the expression of *FGF9*. Importantly, Tan IIA can inhibit the production of inflammatory cytokines and attenuate oxidative stress by modulating the expression of *FGF9.* These results demonstrate the therapeutic potential of using Tan IIA to target the *HAS2*/*FGF9* axis to reduce myocardial damage, offering a viable approach to the treatment of CHD.

## Data Availability

The datasets used and/or analyzed during the current study are available from the corresponding author upon reasonable request.

## Supplementary Materials

Supplementary Materials Supplementary Table 1. Differential expression analysis of 16 key overlapping genes between the control and CAD groups in the GSE23561 dataset.

## References

[1] Fioranelli M., Bottaccioli A. G., Bottaccioli F., Bianchi M., Rovesti M., Roccia M. G. (2018). Stress and inflammation in coronary artery disease: a review psychoneuroendocrineimmunology-based. *Frontiers in Immunology*.

[2] Mortensen M. B., Nordestgaard B. G. (2020). Elevated LDL cholesterol and increased risk of myocardial infarction and atherosclerotic cardiovascular disease in individuals aged 70–100 years: a contemporary primary prevention cohort. *The Lancet*.

[3] Jousilahti P., Vartiainen E., Tuomilehto J., Puska P. (1999). Sex, age, cardiovascular risk factors, and coronary heart disease: a prospective follow-up study of 14 786 middle-aged men and women in Finland. *Circulation*.

[4] Marmot M. G., Bosma H., Hemingway H., Brunner E., Stansfeld S. (1997). Contribution of job control and other risk factors to social variations in coronary heart disease incidence. *The lancet*.

[5] Marmot M. G., Mustard J. F. (2017). Coronary heart disease from a population perspective. *Why are some people healthy and others not?*.

[6] Cleven L., Krell-Roesch J., Nigg C. R., Woll A. (2020). The association between physical activity with incident obesity, coronary heart disease, diabetes and hypertension in adults: a systematic review of longitudinal studies published after 2012. *BMC Public Health*.

[7] Bays H. E., Taub P. R., Epstein E. (2021). Ten things to know about ten cardiovascular disease risk factors. *American journal of preventive cardiology*.

[8] Flora G. D., Nayak M. K. (2019). A brief review of cardiovascular diseases, associated risk factors and current treatment regimes. *Current Pharmaceutical Design*.

[9] Ma Y., Wang Y.-J., Chen B.-R. (2017). Study on association of working hours and occupational physical activity with the occurrence of coronary heart disease in a Chinese population. *PLoS One*.

[10] Qureshi W. T., Zhang Z.-M., Chang P. P. (2018). Silent myocardial infarction and long-term risk of heart failure: the ARIC study. *Journal of the American College of Cardiology*.

[11] Ilcheva L., Risteski P., Tudorache I. (2023). Beyond conventional operations: embracing the era of contemporary minimally invasive cardiac surgery. *Journal of Clinical Medicine*.

[12] Xu H., Li H., Zhu P., Liu Y., Zhou M., Chen A. (2020). Tanshinone IIA ameliorates progression of CAD through regulating cardiac H9c2 cells proliferation and apoptosis by miR-133a-3p/EGFR axis. *Drug Design, Development and Therapy*.

[13] Li W., Gao Z., Guan Q. L. (2023). Tan IIA mitigates vascular smooth muscle cell proliferation and migration induced by ox‐LDL through the miR‐137/TRPC3 axis. *The Kaohsiung Journal of Medical Sciences*.

[14] Wu W. Y., Wang W. Y., Ma Y. L. (2013). Sodium tanshinone IIA silate inhibits oxygen-glucose deprivation/recovery-induced cardiomyocyte apoptosis via suppression of the NF-*κ*B/TNF-*α* pathway. *British Journal of Pharmacology*.

[15] Wang X., Wu C. (2022). Tanshinone IIA improves cardiac function via regulating miR-499-5p dependent angiogenesis in myocardial ischemic mice. *Microvascular Research*.

[16] Lu T. C., Wu Y. H., Chen W. Y., Hung Y. C. (2022). Targeting oxidative stress and endothelial dysfunction using tanshinone IIA for the treatment of tissue inflammation and fibrosis. *Oxidative Medicine and Cellular Longevity*.

[17] Yang F., Sun J., Wu X. (2023). Primary cultures of spermatogonia and testis cells. *Methods in Molecular Biology*.

[18] Yusuf I. O., Chen H.-M., Cheng P.-H. (2021). FGF9 induces neurite outgrowth upon ERK signaling in knock-in striatal Huntington’s disease cells. *Life Sciences*.

[19] Ding W., Ding L., Lu Y. (2023). Circular RNA‐circLRP6 protects cardiomyocyte from hypoxia‐induced apoptosis by facilitating hnRNPM‐mediated expression of FGF‐9. *FEBS Journal*.

[20] Zhu X., Deng X., Huang G. (2014). A novel mutation of Hyaluronan synthase 2 gene in Chinese children with ventricular septal defect. *PLoS One*.

[21] Lin Z., Fan W., Yu X., Liu J., Liu P. (2022). Research into the mechanism of intervention of SanQi in endometriosis based on network pharmacology and molecular docking technology. *Medicine (Baltimore)*.

[22] Zhao J., Jiao W., Sui X., Zou J., Wang J., Lin Z. (2023). Construction of a prognostic model of luteolin for endometrial carcinoma. *Journal of Translation Research*.

[23] Lin Z., Sui X., Jiao W., Chen C., Zhang X., Zhao J. (2022). Mechanism investigation and experiment validation of capsaicin on uterine corpus endometrial carcinoma. *Frontiers in Pharmacology*.

[24] Amran M. S., Bahar N. B., Akash S. (2022). Physiology and pathology of the cardiovascular system. *Cardiovascular Diseases*.

[25] Wang S., Li Y., Jiang C., Tian H. (2018). Fibroblast growth factor 9 subfamily and the heart. *Applied Microbiology and Biotechnology*.

[26] Sun J., Wang Z., Shi H., Gu L., Wang S., Wang H. (2020). LncRNA FAF inhibits fibrosis induced by angiotensinogen II via the TGF*β*1-P-Smad2/3 signalling by targeting FGF9 in cardiac fibroblasts. *Biochemical and Biophysical Research Communications*.

[27] Said S. S. (2016). *Controlled Delivery of Angiogenic and Arteriogenic Growth Factors from Biodegradable Poly (Ester Amide) Electrospun Fibers for Therapeutic Angiogenesis*.

[28] Ng A., Wong M., Viviano B. (2009). Loss of glypican-3 function causes growth factor-dependent defects in cardiac and coronary vascular development. *Developmental Biology*.

[29] Addissouky T. A., El Tantawy El Sayed I., Ali M. M. (2024). Shaping the future of cardiac wellness: exploring revolutionary approaches in disease management and prevention. *Journal of Clinical Cardiology*.

[30] Zhu P.-C., Shen J., Qian R.-Y. (2023). Effect of tanshinone IIA for myocardial ischemia/reperfusion injury in animal model: preclinical evidence and possible mechanisms. *Frontiers in Pharmacology*.

[31] Xu Q., Wang Z., You S., Yuan L., Li H. (2023). Mechanism for effect of tanshinone IIA on alleviating cardiomyocyte injury induced by oxygen glucose deprivation. *Zhong nan da xue xue bao Yi xue ban= Journal of Central South University Medical Sciences*.

[32] Deng H., Yu B., Li Y. (2021). Tanshinone IIA alleviates acute ethanol‐induced myocardial apoptosis mainly through inhibiting the expression of PDCD4 and activating the PI3K/Akt pathway. *Phytotherapy Research*.

[33] Ma X., Zhang L., Gao F., Jia W., Li C. (2023). Salvia miltiorrhiza and Tanshinone IIA reduce endothelial inflammation and atherosclerotic plaque formation through inhibiting COX-2. *Biomedicine & Pharmacotherapy*.

[34] Ren Z., Tong Y., Xu W., Ma J., Chen Y. (2010). Tanshinone II A attenuates inflammatory responses of rats with myocardial infarction by reducing MCP-1 expression. *Phytomedicine*.

[35] Xu L., He D., Wu Y., Shen L., Wang Y., Xu Y. (2022). Tanshinone IIA inhibits cardiomyocyte apoptosis and rescues cardiac function during doxorubicin-induced cardiotoxicity by activating the DAXX/MEK/ERK1/2 pathway. *Phytomedicine*.

[36] Petz A., Grandoch M., Gorski D. J. (2019). Cardiac hyaluronan synthesis is critically involved in the cardiac macrophage response and promotes healing after ischemia reperfusion injury. *Circulation Research*.

[37] Lagendijk A. K., Goumans M. J., Burkhard S. B., Bakkers J. (2011). MicroRNA-23 restricts cardiac valve formation by inhibiting Has2 and extracellular hyaluronic acid production. *Circulation Research*.

[38] Azizidoost S., Farzaneh M. (2023). MicroRNAs as a novel player for differentiation of mesenchymal stem cells into cardiomyocytes. *Current Stem Cell Research and Therapy*.

[39] Sun Y., Ying X., Li R., Weng M., Shi J., Chen Z. (2022). FGF9 promotes expression of HAS2 in palatal elevation via the wnt/*β*-catenin/TCF7L2 pathway. *Biomolecules*.

[40] Soares R. O., Losada D. M., Jordani M. C., Évora P., Castro-e-Silva O. (2019). Ischemia/reperfusion injury revisited: an overview of the latest pharmacological strategies. *International Journal of Molecular Sciences*.

[41] Maxwell S. R., Lip G. Y. (1997). Reperfusion injury: a review of the pathophysiology, clinical manifestations and therapeutic options. *International Journal of Cardiology*.

[42] Zhu Y., Xian X., Wang Z. (2018). Research progress on the relationship between atherosclerosis and inflammation. *Biomolecules*.

[43] Raggi P., Genest J., Giles J. T. (2018). Role of inflammation in the pathogenesis of atherosclerosis and therapeutic interventions. *Atherosclerosis*.

[44] Hanna A., Frangogiannis N. G. (2020). Inflammatory cytokines and chemokines as therapeutic targets in heart failure. *Cardiovascular Drugs and Therapy*.

[45] Bartekova M., Radosinska J., Jelemensky M., Dhalla N. S. (2018). Role of cytokines and inflammation in heart function during health and disease. *Heart Failure Reviews*.

[46] Xing J., Cai H., Lin Z. (2023). Examining the function of macrophage oxidative stress response and immune system in glioblastoma multiforme through analysis of single-cell transcriptomics. *Frontiers in Immunology*.

[47] Lin Z., Li X., Shi H. (2024). Decoding the tumor microenvironment and molecular mechanism: unraveling cervical cancer subpopulations and prognostic signatures through scRNA-Seq and bulk RNA-seq analyses. *Frontiers in Immunology*.

