# Supplementary material for: Tanshinone IIA Protects Ischemia/Reperfusion-Induced Cardiomyocyte Injury by Inhibiting the HAS2/FGF9 Axis
Source: Cardiol Res Pract. 2024 Nov 13;2024:2581638. doi: 10.1155/2024/2581638 (PMC11578662; doi:10.1155/2024/2581638)
Supplement: Supplementary Materials — Supplementary Table 1. Differential expression analysis of 16 key overlapping genes between the control and CAD groups in the GSE23561 dataset. [file 2581638.f1.pdf]

Supplementary Table 1. Differential expression analysis of 16 key overlapping genes between the control and CAD groups in the GSE23561 dataset.

Gene	P.Value	logFC
A1BG	5.10E-03	1.029148
ABCA1	3.51E-03	1.028248
ABCB11	5.41E-03	1.004704
ACTN4	4.89E-04	1.061836
ADAM12	4.29E-03	1.321874
APCS	1.21E-03	1.16512
CETN1	5.20E-04	1.084033
CFB	3.75E-04	1.045549
CYP8B1	3.52E-03	1.078904
DAG1	7.37E-03	1.155842
FDFT1	2.94E-03	1.171705
FGF9	6.55E-04	1.13964
HBA1	2.07E-03	1.40285
HBA2	2.07E-03	1.40285
HBB	2.07E-03	1.40285
LAMA3	1.74E-03	1.28437

Group	A1BG	ABCA1	ABCB11	ACTN4	ADAM12	APCS	CETN1	CFB	CYP8B1	DAG1	FDFT1	FGF9	HBA1	HBA2	HBB	LAMA3
disease state: Control	0.23	0.04	0.06	0.05	0.02	0.03	0.04	0.06	0.09	0.04	0.13	0.04	17.78	17.78	17.78	0.04
disease state: Control	0.22	0.08	0.09	0.06	0.05	0.05	0.06	0.07	0.07	0.07	0.05	0.06	26.04	26.04	26.04	0.04
disease state: Control	0.28	0.06	0.07	0.07	0.06	0.04	0.04	0.06	0.05	0.06	0.04	0.04	26.63	26.63	26.63	0.04
disease state: Control	0.06	0.03	0.02	0.12	0.01	0.02	0.06	0.04	0.03	0.01	0.08	0.04	7.86	7.86	7.86	0.01
disease state: Control	0.18	0.08	0.12	0.08	0.07	0.07	0.1	0.1	0.17	0.08	0.07	0.06	38.29	38.29	38.29	0.07
disease state: Control	0.08	0.07	0.06	0.09	0.05	0.05	0.07	0.07	0.13	0.07	0.17	0.05	16.71	16.71	16.71	0.05
disease state: Control	0.19	0.11	0.09	0.08	0.09	0.09	0.1	0.09	0.1	0.1	0.07	0.11	28.13	28.13	28.13	0.08
disease state: Control	0.17	0.07	0.07	0.07	0.05	0.05	0.07	0.07	0.07	0.06	0.16	0.07	23.45	23.45	23.45	0.05
disease state: Control	0.22	0.04	0.06	0.09	0.03	0.05	0.07	0.09	0.12	0.06	0.2	0.04	15.26	15.26	15.26	0.04
disease state: Coronary	0.48	0.07	0.11	0.1	0.05	0.06	0.15	0.14	0.2	0.07	0.21	0.07	25.33	25.33	25.33	0.07
disease state: Coronary	0.41	0.09	0.09	0.2	0.08	0.09	0.09	0.11	0.14	0.11	0.18	0.09	40.3	40.3	40.3	0.08
disease state: Coronary	0.22	0.15	0.15	0.25	0.19	0.14	0.15	0.17	0.18	0.16	0.16	0.16	148.17	148.17	148.17	0.14
disease state: Coronary	0.35	0.11	0.11	0.16	0.09	0.09	0.13	0.12	0.13	0.1	0.26	0.11	29.09	29.09	29.09	0.08
disease state: Coronary	0.26	0.2	0.15	0.15	0.14	0.15	0.14	0.16	0.17	0.16	0.17	0.16	121.42	121.42	121.42	0.14
disease state: Coronary	0.36	0.16	0.2	0.14	0.11	0.12	0.18	0.18	0.25	0.14	0.33	0.15	46.73	46.73	46.73	0.12
